# Advances in Development of New Treatment for Leishmaniasis

**DOI:** 10.1155/2015/815023

**Published:** 2015-05-11

**Authors:** Juliana Perrone Bezerra de Menezes, Carlos Eduardo Sampaio Guedes, Antônio Luis de Oliveira Almeida Petersen, Deborah Bittencourt Mothé Fraga, Patrícia Sampaio Tavares Veras

**Affiliations:** Laboratório de Patologia e Biointervenção, CPqGM, FIOCRUZ, Rua Waldemar Falcão 121, Candeal, 40296-710 Salvador, BA, Brazil

## Abstract

Leishmaniasis is a neglected infectious disease caused by several different species of protozoan parasites of the genus *Leishmania*. Current strategies to control this disease are mainly based on chemotherapy. Despite being available for the last 70 years, leishmanial chemotherapy has lack of efficiency, since its route of administration is difficult and it can cause serious side effects, which results in the emergence of resistant cases. The medical-scientific community is facing difficulties to overcome these problems with new suitable and efficient drugs, as well as the identification of new drug targets. The availability of the complete genome sequence of *Leishmania* has given the scientific community the possibility of large-scale analysis, which may lead to better understanding of parasite biology and consequent identification of novel drug targets. In this review we focus on how high-throughput analysis is helping us and other groups to identify novel targets for chemotherapeutic interventions. We further discuss recent data produced by our group regarding the use of the high-throughput techniques and how this helped us to identify and assess the potential of new identified targets.

## 1. Leishmaniasis Treatment

The World Health Organization (WHO) considers leishmaniasis to be one of the main neglected diseases in the world, affecting primarily the poor population of underdeveloped and developing countries [1]. Infection by parasites of the genus* Leishmania* causes a broad spectrum of clinical manifestations including subclinical (inapparent), localized (skin lesions), and disseminated infections (cutaneous, mucosal, or visceral) [2–5].

Tropical diseases, which are globally disperse and of great social-economic impact, affect mostly poor people in developing countries. Therefore, commercial interest in developing new pharmaceutical compounds [6, 7] for these diseases is limited because their treatment needs to be affordable to ensure access by the affected poor population. In addition, the advances in our understanding of the biology of* Leishmania* spp. have not translated into effective new chemotherapeutic compounds [8].

Recently, few alternative drugs have emerged for the treatment of leishmaniasis (Tables 1 and 2). None of the available drugs can be considered ideal due to their high toxicity, long duration of treatment, and severe adverse reactions, which often lead to treatment abandonment. In addition, the most commonly used drugs do not eliminate the parasites completely from all infected individuals [9, 10].

Pentavalent antimonials are the most frequently used drugs for the treatment of leishmaniasis, despite their variable effectiveness for both visceral and cutaneous leishmaniasis [1, 11–14]. Due to accumulation in the tissues, antimonials can cause serious adverse effects, such as vomiting, nausea, anorexia, myalgia, abdominal pain, headache, arthralgia, and lethargy and can rarely cause the severe reaction of fatal cardiac arrhythmia [1, 12, 15–18]. These adverse effects are due to severe cardiotoxicity, pancreatitis, and nephrotoxicity that can require hospitalization and close monitoring of patients [10, 13]. Efforts to reduce the toxicity of these drugs have not been effective [19]. The adverse effects and the lengthy treatment period lead to treatment noncompliance and abandonment, favoring the emergence of resistant* Leishmania* strains, as in Bihar (India) [20].

Despite its high toxicity, amphotericin B is one of the first-line drugs for leishmaniasis treatment [21, 22]. Its intravenous administration frequently causes rigor, chills, and fever, associated with myocarditis and nephrotoxicity [1, 15, 16]. Amphotericin B formulations (the lipid complex, colloidal form, and the liposomal form) were developed to reduce adverse effects and improve pharmacokinetics and bioavailability. Although proven less toxic, these alternative formulations of amphotericin B have limitations for use in developing countries: they are very costly and unstable at higher temperatures (requiring cooling) [22, 23].

Pentamidine, another drug currently used for leishmaniasis treatment, is highly toxic and triggers important adverse effects, such as diabetes mellitus, severe hypoglycemia, hypotension, myocarditis, and renal toxicity and can ultimately cause death [1, 24]. This drug is currently used infrequently due to the appearance of resistance cases [25], its high toxicity, and low efficacy [26]. Currently, pentamidine is mainly recommended when used in combined therapeutic protocols [16, 20].

Paromomycin is an alternative drug for leishmaniasis treatment [1]; however, parenteral formulations can cause serious adverse reactions, including nephrotoxicity and ototoxicity and more rarely hepatotoxicity [27–29]. Mitelfosine, an alternative antileishmanial drug [1, 30, 31], has the advantage of oral administration. However, its use is limited for pregnant women, since the most severe side effects of this drug are the induction of teratogenesis and the occurrence of a high index of treatment failure [22, 32]. In the last 35 years, many compounds have been found to have low efficacy against leishmaniasis, including rifampicin, tamoxifen, doxycycline, monomycine, trimethoprim, and nifurtimox [16, 21, 24, 33–44].

Regarding selective antileishmanial activity, pentavalent antimonials can act against different species of* Leishmania*, whereas pentamidine has limited activity against specific* Leishmania* species [40]. Paromomycin [45] can be used topically to treat localized leishmaniasis caused by* L. braziliensis*, but not for that caused by other species of* Leishmania* [46, 47]. Miltefosine, which is effective for the treatment of visceral leishmaniasis caused by* L. donovani*, seems to be ineffective in the treatment of infection by* L. major* and* L. braziliensis* [45]. Because many current antileishmanial drugs have only species-specific activity, new drugs or protocols should be scrutinized to determine their activity for the entire spectrum of* Leishmania* species and diseases.

Alternative protocols, such as the rational combination of drugs, have favorably reduced individual doses, treatment duration, and adverse effects [22]. In addition, these protocols provide a short-term solution by reducing costs, the frequency of treatment failure, and the occurrence of drug resistance [40, 48]. Seifert and Croft [49] evaluated a combination of antileishmanial drugs as an alternative to be used in place of pentavalent antimonials in cases of visceral leishmaniasis resistant to these drugs. The combination of miltefosine with amphotericin B or paromomycin was very efficient and could be helpful to treat antimony-resistant VL infections. Sundar and collaborators [48] compared the efficacy and safety of the treatment combining three antileishmanial drugs (amphotericin B in liposomal formulation, miltefosine, and paromomycin), to those of monotherapy with amphotericin B in a study conducted in India. Although the efficacy of this multidrug regimen was similar to the monotherapy, the authors observed less frequent adverse effects, less severe disease, and shorter duration of the treatment with the multidrug regimen. Phase 3 studies have also been conducted in Asia and Africa to investigate the effectiveness of multidrug treatment protocols for visceral leishmaniasis. Whether combination therapy will delay resistance, and how this is best achieved, will only be known from long-term studies [10]. In addition, combinations of immunotherapy and antileishmanial drugs have been investigated in the last 10 years [10, 50, 51], showing variable efficacy.

Another approach for alternative treatment of leishmaniasis is the use of controlled release systems, such as liposomes and nanoparticles. This type of systems provides a greater efficacy and safety once drugs are adsorbed or encapsulated in carriers, reducing the dose and adverse reactions of conventional formulations [52–54]. A liposomal formulation of amphotericin B, Ambisome, has been used to treat leishmaniasis, showing better results than that obtained using the sodium stibogluconate. It produced fewer adverse reactions and treatment failures in patients with cutaneous leishmaniasis [55, 56]. Ambisome has been used to treat HIV-*Leishmania* coinfected patients; however, this treatment did not reduce relapse and mortality rates for these coinfected patients, compared to those rates in HIV negative* Leishmania*-infected patients [57]. Similar results were described by Ritmeijer et al. [58] in a study conducted in Ethiopia. The stearylamine liposome formulation containing sodium stibogluconate developed by Roychoudhury et al. [59] showed efficacy against strains of* L. donovani* resistant to sodium stibogluconate. Shio et al. [60] revealed that another drug, oleylphosphocholine (OlPC), formulated as liposomes, killed intracellular amastigotes of* L. major* and* L. mexicana* in macrophages. Using a tattoo machine, they injected this formulation directly into the cutaneous lesions of infected BALB/c mice, resulting in a complete regression of the skin lesions. However, further studies are needed to evaluate this new treatment approach.

Functionalized carbon nanotubes also have been tested as drug carriers against leishmaniasis. Amphotericin B attached to functionalized carbon nanotubes has a significantly greater leishmanicidal activity than conventional amphotericin B in* L. donovani*-infected hamsters [61]. Similarly, the attachment of betulin, a pentacyclic triterpenoid, to functionalized carbon nanotubes improved its leishmanicidal effect, with lower toxicity [62]. Recently, Ribeiro et al. [63] showed that amphotericin B conjugated to nanoparticles composed of chitosan-chondroitin sulfate had less cellular toxicity than conventional amphotericin B formulation and was more prone to kill* Leishmania* parasites inside parasitophorous vacuoles. To address the efficacy of these new antileishmanial formulations, additional* in vivo* studies should be conducted.

Another strategy to improve antileishmanial treatment is the identification of new targets both in parasites and in host cells. New potential targets for drugs have been identified in molecular and biochemical studies and some have been validated [64–66]. Studies to better understand the biology of host-parasite interactions would facilitate the design of more effective drugs against* Leishmania* infection. High performance techniques are currently being employed to identify particular parasite and host cell expressed molecules that can be finally used as chemotherapeutic targets [64–66]. Indeed, transcriptomics and proteomics have been important large-scale tools for understanding the biology of* Leishmania* and host interaction, favoring the identification of new targets for leishmaniasis treatment [67, 68].

## 2. High-Throughput Screening for New Targets

The robustness of critical cellular metabolism relies on a complex and highly linked adjustable network with redundant or alternative pathways to maintain the usual flow of molecules and materials in the cell [83]. Therefore, targeting parasite and host cell pathways, selected using high-throughput analyses, appears more promising than focusing on a specific enzyme or other individual molecules [84]. The use of a target-based drug discovery approach that inhibits an individual target molecule would seldom generate the desired outcome. On the other hand, if a critical pathway is the target, then chemical compounds that interfere in this pathway could be selected. Apparently, studies of complex living systems should recognize with the limitations of more simple systems to develop effective and safe drugs to control diseases [85]. Therefore, consideration of the complexity of large-scale data that is central to drug discovery and development in the postgenomic era is a difficult undertaking [84]. Recently, these complex technologies have emerged as important tools for understanding the mechanisms of disease establishment, resistance to pathogens [86–88], and searching for new drug targets [84].

The massive increase in genomic data for pathogens that cause tropical diseases, in particular the completion of their genome sequence, has provided the opportunity to identify novel vaccine and chemotherapeutic targets. The development of functional genomics tools, such as microarray and more recently deep sequencing technology, as well as proteomics, has revealed strategies to achieve clinical goals [89]. In addition, these advances allowed the emergence of bioinformatics in the postgenomic era that enormously hastened the research process. Furthermore, computational algorithms and multiple confirmatory assay formats combined with high-throughput screening methodologies greatly contribute to the identification and characterization of novel potent drug targets [67]. Among large-scale strategies, transcriptomic profiling and proteomics have emerged as potential approaches to these final goals [67]. Proteomics in addition to the transcriptomics approach, performed in several laboratories, have identified numerous stage-specific genes in* Leishmania* spp. [90–96], as well as primary resistance mechanisms and novel parasite targets [95, 97–99]. Between these two approaches, proteomics has proven more advantageous than microarray for the discovery of new therapeutic strategies in diseases caused by pathogens (Table 3) [69–82]. In particular, for trypanosomatids, proteomics has been preferred, because mRNA has been found to correlate poorly with protein species derived from the same gene [89].

Proteomics is being widely employed to study* Leishmania* and, in association with the annotated sequenced genome of* Leishmania*, seems a valuable strategy for dissection of both protein expression/regulation and function. Expression proteomics exploits the differential expression of leishmanial proteins as biomarkers for early diagnosis [100]. Furthermore, immunoproteomics efforts focused on evaluating responses to defined parasite T-cell epitopes as vaccine/diagnostic targets. These approaches have also expanded the understanding of the array of events involved in* Leishmania* infection [86, 87]. The construction of an index map for a large number of* Leishmania* species is another large-scale approach that was exploited by several authors and contributed to a better understanding of the biology of this parasite, aiming to identify and locate as many parasite proteins as possible [21, 101]. Microarray technology has also been used successfully to identify critical genes expressed during the development and differentiation of* L. donovani* [96, 102–104] and* L. infantum* [89, 105] parasites. Recently, using large-scale approaches, many groups have identified developmentally regulated proteins by comparing expression patterns of soluble and whole cell lysate proteins of either* L. donovani* and* L. infantum in vitro* axenic promastigotes, as well as induced axenic amastigote forms [95, 97, 105].

Drug screening assays are available for promastigotes, axenic, and intracellular amastigote forms and for parasite infected animal models. Intracellular amastigotes, the parasite form adapted to live inside host cells, are the ideal sample for high-throughput screening assays [8]. A recent study used an automated high-throughput screening assay to discover new antileishmanial compounds and brought promising candidates to the leishmaniasis drug discovery pipeline [65]. In addition, the same group elegantly applied an image-based high-throughput approach and developed computer-assisted algorithms to interpret the infection images and quantify the activities of the antiparasitic compounds [106].

Because leishmaniasis management relies on drug treatment, drug resistant parasites are a major challenge to this field. However, the mechanisms responsible for drug resistance are only partially understood. Therefore, to elucidate the molecular mechanisms of drug resistance, several studies examined differences in protein expression pattern between drug susceptible and resistant parasites using comparative proteomics [68]. Specifically, proteomic screening was employed to evaluate drug resistance to methotrexate and antimony in* L. major*,* L. donovani,* and* L. infantum* [98, 107–110]. These studies also contributed to a better understanding of the parasite biology and its response to treatment.

Our group used the proteomic approach to search for new chemotherapeutic leishmanial targets and to modulate these targets to control* Leishmania* infection. In this study, CBA mouse model of cutaneous leishmaniasis [111] that is highly susceptible to infection* in vivo* with* L. amazonensis* was used. In addition, macrophages from the same strain of mice are permissive to* L. amazonensis* infection* in vitro* [112]. Proteomic analysis was performed to search for new targets with potential for chemotherapeutic intervention in these macrophages infected with* L. amazonensis* [113]. Thereby, we identified a set of proteins with modulated expression in macrophages infected with* L. amazonensis* [113]. Our strategy was to select from among these modulated proteins those with the potential to be modulated by drugs that could act not only on the target present in the host cell, but also on targets present in the parasite. Among them, we selected the transcription factor hypoxia-inducible factor- (HIF-) 1*α* for study. This transcription factor is one of the client proteins for the heat shock protein 90 (HSP90), which is a molecular chaperone that is highly conserved among organisms from different kingdoms, such as bacteria, yeast, and eukaryotic cells [114]. Notably, HSP90 is expressed both in macrophages and in* Leishmania* [115, 116]. Some molecules with anticancer activity that act against HIF-1*α* actually act against HSP90. Below, we will further discuss evidence of the antileishmanial effect of chemicals that modulate HSP90.

## 3. HSP90

The heat shock protein 90 (HSP90) is ubiquitously expressed throughout all kingdoms, except Archaea [115, 116]. Its main function is to serve as molecular chaperone helping the correct folding of nascent proteins, avoiding miss-folding and protein aggregate formation. This molecular chaperone is expressed in physiological conditions, accounting as much as 2% of the total soluble proteins in the cell and up to 10% in cells under stress [117–120]. HSP90 functions as a dimeric ATP-dependent chaperone. The ATP-binding pocket is located in the N-terminal site of the protein; the middle domain is used to interact with client protein and cochaperones while the C-terminal is used in the dimerization process. The ATP-biding site at the N-terminal region of the HSP90 can be specifically inhibited upon by ansamycin-benzoquinone antibiotics, such as the geldanamycin (GA), its derivatives, and other small molecules. GA and other HSP90 inhibitors act by competing with high affinity against the ATP for the ATP-ase pocket at the N-terminal domain of the HSP90 [121, 122]. Once the ATP-ase activity of the HSP90 is shut, nascent, unfolded, misfolded, and client proteins are ubiquitinated and degraded through the proteasome pathway [123]. Since HSP90 controls hundreds of proteins expression including several oncoproteins, many HSP90 inhibitors have been tested as anticancer drugs including the 17-(allylamino)-17- demethoxygeldanamycin (17-AAG). Several of these compounds have anticancer activity and entered clinical trials as candidates for treatment of different human cancers [124].

Besides of the anticancer activity of those HSP90 inhibitors, some of them have been tested* in vitro* and* in vivo* against infectious diseases caused by* Plasmodium*,* Trypanosoma*, and* Toxoplasma* with promising results in the majority of the cases [125–128]. In protozoans, several regulatory proteins, such as tyrosine-kinases, cytoskeletal proteins, histones, transcription factors, and DNA polymerases, require HSP90 interaction in order to complete their folding process [129, 130]. In* Leishmania* spp., HSP90 client proteins play essential roles in cell cycle control, cellular signal transduction, and transcription regulation. Inhibition of HSP90 from promastigotes of* L. donovani* induced shape change into rounded cells and reduction of the flagellum, cell cycle arrest in G2 phase, and expression of A2 proteins, typical of amastigote stage of* Leishmania* parasites [131].

Recently, we have conducted a series of experiments to determine the effect of HSP90 pharmacologic inhibition on the outcome of the infection caused by* L. amazonensis*. So far, we have tested three different HSP90 inhibitors, GA, 17-AAG, and radicicol (RD). We obtained similar* in vitro* results regarding intracellular parasite killing [132].

First, we assessed the ability of 17-AAG to kill extracellular promastigote parasites. We observed, by direct counting, that 17-AAG inhibits* L. amazonensis* growth at a nanomolar range (65 ± 7 nM). We also showed that 17-AAG is able to reduce the percentage of macrophage infection and the parasite burden in a time-dose dependent manner with an EC_50_ of 149 ± 7 nM. On the other hand, the dose able to reduce macrophage viability by 50% (CC_50_) is 10,830 ± 1,700 nM, as assessed by Alamar Blue assay. As result, we show that 17-AAG has a selective index (SI) of 72.68. This means that 17-AAG is 72 times more efficient against intracellular* Leishmania* than the macrophage host cell. These results are very promising since high doses used in cancer patients from clinical trials might not be needed for the treatment of leishmaniases or other parasitic diseases [132, 133].

Another important observation is the fact that 17-AAG is able to kill both promastigotes and amastigotes forms of the* Leishmania*. Similarly to the effect on promastigote forms and on early-phagocytized parasites and on intracellular completed differentiated amastigote, 17-AAG causes parasite death in a dose-time dependent manner. This is an essential observation since the amastigote is the persistent form found in mammalian host cells [134]. These results were the final step before the use of 17-AAG in the murine models of leishmaniases. To date, we have tested 17-AAG in a* L. braziliensis*, BALB/c murine model. 17-AAG, in this model, has proven to be efficient, reducing the lesion size and parasite burden after intraperitoneal treatment. However, draining lymph nodes did not have their parasite burden reduced by the treatment [133].

We also observed that treatment of infected macrophages with 17-AAG actually reduces proinflammatory cytokines and chemokines like TNF-alpha and MCP-1, known to play a role in parasite clearance. 17-AAG treatment also inhibits superoxide and nitric oxide (NO) production by infected macrophages, both known as leishmanicidal molecules [132, 133]. Previous data from the literature supports the notion that 17-AAG and other HSP90 inhibitors actually act as an anti-inflammatory molecule in different models [135–137]. Reduced NO production might be explained by the fact that iNOS itself is a client protein of HSP90 and it has been shown that HSP90 inhibition reduces NO production in other models [138, 139]. This might provide 17-AAG with an advantage, since in lesions of patients with cutaneous and mucocutaneous leishmaniasis intense inflammatory response is observed with exacerbated IFN-gamma, TNF-alpha, and oxidative response that causes tissue damage.

We also observed that 17-AAG induces intense parasite vacuolization with autophagic features [132]. To date, it seems that autophagy plays a role in parasite death, but it is hard to determine if* Leishmania* parasite is suffering an autophagic cell death [119]. To prove that it would be necessary to block the autophagic pathway and observe an increase in parasite viability after 17-AAG treatment. Although autophagy is required for housekeeping processes and promastigote to amastigote differentiation in* Leishmania*, it would be interesting to investigate the role autophagy actually plays in* Leishmania* death caused by 17-AAG.

It is clear that HSP90 plays an important role in parasite housekeeping, metabolism, and cell cycle, particularly considering the huge number of client proteins that are under HSP90 control. Our data support the idea that HSP90 serves as a major molecular target for chemotherapy intervention in parasitic diseases especially in leishmaniases. The main advantage of HSP90 inhibition for leishmaniasis treatment is the possibility of attacking several parasite regulatory proteins with a single drug. Besides that, the treatment range of nanomolar and a SI of 72.68 are very promising, especially considering that 17-AAG and other HSP90 inhibitors have been or are being tested as anticancer drugs in clinical trials. In sum, these results indicate that HSP90 is an interesting molecular target that should be more explored specially regarding parasitic disease treatment. In addition, our findings support the notion that 17-AAG as well as other HSP90 inhibitors are promising antileishmanial drugs that could be used alone or as synergistic drugs with the aim of reducing toxicity and resistance and increase potency [132, 133].

## 4. Concluding Remarks

The search for new antileishmanials is due to the lack in the available drugs for leishmaniasis treatment as they can have high toxicity, be used a long term, and cause severe adverse reactions. This often leads to treatment abandonment and failure. In addition, many current antileishmanials do not eliminate the parasites completely from all infected individuals and have only species-specific activity. Alternative protocols, such as the rational combination of drugs or combinations of immunotherapy and antileishmanial drugs, have favorably reduced individual doses, treatment duration, and adverse effects. However, comprehensive long-term studies need to be developed to determine the actual efficacy of these alternative protocols. Controlled release systems, such as liposomes and nanoparticles, provide a greater efficacy and safety once drugs are adsorbed or encapsulated in carriers, reducing the dose and adverse reactions of conventional formulations. In addition, to address the efficacy of these new antileishmanial formulations,* in vivo* studies should also be conducted.

In order to identify new chemotherapeutic targets for control of* Leishmania* infection, high-throughput studies have proven to be useful. Between microarray and proteomics approaches, data in the literature support the idea that proteomics is superior to microarray for the discovery of new therapeutic strategies in diseases caused by pathogens, being also widely employed to study* Leishmania* for screening of drug resistance in various* Leishmania* species. Our group using proteomics was able to identify among a set of proteins in infected macrophages new targets with potential for chemotherapeutic intervention. As described above, we demonstrated that chemicals that inhibit one of these targets have shown a potent antileishmanial effect.

## Figures and Tables

**Table 1:** Drugs used for the treatment of leishmaniasis.

Drugs	Administration route	Dosage	Efficacy	Toxicity
Pentavalent antimonials [1, 10–20]	IM, IV, or IL	20 mg/kg/day (28–30 days)	35–95% (depending on area)	Severe cardiotoxicity, pancreatitis, nephrotoxicity, hepatotoxicity

Amphotericin B [1, 15, 16, 21–23, 40]	IV	0.75–1 mg/kg/day (15–20 days, daily or alternately)	>90%	Severe nephrotoxicity, infusion-related reactions, hypokalemia, high fever

Liposomal amphotericin B [22, 23, 55–58]	IV	10–30 mg/kg total dose (single dose 3–5 mg/kg/dose)	>97%	Mild rigors and chills during infusion Mild nephrotoxicity (infrequent and mild)

Miltefosine [1, 16, 30–32, 45, 48, 49]	Oral	100–150 mg/day (28 days)	Asia: 94% (India); Africa: 60%–93%	Vomiting and diarrhoea, nephrotoxicity, hepatotoxicity, teratogenicity

Paromomycin [1, 27–29, 45]	IM (VL) or topic (CL)	15 mg/day (21 days) or 20 mg/kg (17 days)	94% (India) 46–85% (Africa)	Severe nephrotoxicity, ototoxicity, hepatotoxicity

Pentamidine [1, 16, 20, 24–26]	IM	3 mg/kg/day IM every other day for 4 injections	35–96% (depending on *Leishmania* species)	High rate of hyperglycemia, as a result of pancreatic damage; hypotension, tachycardia, and electrocardiographic changes

IV: intravenous administration; IM: intramuscular administration; IL: intralymphatic administration.

**Table 2:** Advantages and disadvantages of drugs used for the treatment of leishmaniasis.

Drugs	Advantages	Disadvantages	Resistance	Price	Comment
Pentavalent antimonials [1, 10–20]	Easily availability and low cost	Quality control; length of treatment; painful injection; toxicity; resistance in India	Common (>65% in Bihar, India)	$50–198	First line drugs but with high incidences of resistance; variable response in different species that cause CL

Amphotericin B [1, 15, 16, 21–23, 40]	Primary resistance is unknown	Need for slow intravenous infusion; dose-limiting nephrotoxicity; heat instability	Laboratory strains	~$21–100	Severe toxicity; need for prolonged hospitalization; first-line drug for VL in India, where there is antimonial resistance

Liposomal amphotericin B [22, 23, 55–58]	Highly effective; low toxicity	Price; need for slow intravenous infusion; heat stability (needs to be stored below 25°C)	Not documented	$280–3000	High cost

Miltefosine [1, 16, 30–32, 45, 48, 49]	Effective and safe	Price; possibly teratogenic; potential for resistance (half-life); poor patient compliance	Laboratory strains	$70–150	Effective orally but its long half-life may encourage emergence of resistance on prolonged use; effective for VL and against some species that cause CL; contraindicated in pregnancy as found to be teratogenic in rats

Paromomycin [1, 27–29, 45]	Effective, well tolerated, and relatively cheap	Efficacy varies between and within regions; potential for resistance	Laboratory strains	$10–15	Low cost; lack of efficacy in East Africa; topical formulation available for CL

Pentamidine [1, 16, 20, 24–26]	Short-time course	Efficacy varies between *Leishmania* species	Not documented	—	For specific forms of CL in South America only; first line of treatment of CL in French Guiana

**Table 3:** High-throughput strategies to identify targets in several diseases of different causes.

Disease/pathogen	Use of high throughput	Main result
*Leishmania infantum chagasi *	Mechanisms involved in parasite resistance to treatment	Identification of 32 differentially expressed proteins in miltefosine sensitive and resistant parasites using comparative proteomics [69]

*Leishmania infantum *	Mechanisms involved in parasite resistance to treatment	Identification of 97 differentially expressed proteins in amphotericin B-sensitive and -resistant parasites using quantitative proteomics [70]

*Trypanosoma cruzi *	Mechanisms involved in parasite resistance to treatment	Identification of proteins involved in the effect of naphthoimidazoles N1, N2 and N3 on the parasite using proteomics [71]

*Trypanosoma cruzi *	Mechanisms of drug action and resistance	Identification of proteins that could be related to benznidazole reductive activation and/or resistance mechanisms [72]

*Trypanosoma brucei *	Drug development	Proteomics study showing that 2,4-diaminopyrimidines have a good *in vitro* and *in vivo* pharmacological profile against trypanosomatid protozoans [73]

*Toxoplasma gondii *	Mechanisms involved in parasite resistance to treatment	First proteomics insights into sulfadiazine resistance in *T. gondii* resistant strains isolated from clinical cases [74]

*Plasmodium falciparum *	Drug development and mechanisms of drug action	Proteomics study showing that indolone-N-oxide causes a profound destabilization of the malaria-infected erythrocytes membrane through a mechanism apparently triggered by the activation of a redox signaling pathway rather than direct oxidative damage [75]

*Plasmodium falciparum *	Mechanisms involved in parasite resistance to treatment	Identification of a specific response to doxycycline treatment, involving mitochondrion and apicoplast [76]

*Mycobacterium tuberculosis *	Identification of markers of treatment response	Identification of a nonculture based, five-marker signature predictive of 8-week culture status using proteomics [77]

*Neisseria gonorrhoeae *	Drug resistance and mechanisms of drug action	Comparative proteomics study providing knowledge of the mode of action of antibiotic and secondary target proteins implicated in adaptation and compensatory mechanisms [78]

*Staphylococcus aureus *	Drug development and mechanisms of drug action	Proteomics study showing that MntABC might be a potential therapeutic target for the development of antibiotics and that *in vivo* proteomics data will serve as a valuable basis for defining potential antigen combinations for multicomponent vaccines [79]

Cancer	Drug development and mechanisms of drug action	First proteomic analysis regarding Aubipy_c_ cytotoxicity in A2780/S ovarian cancer cell line showing that Aubipy_c_ treatment affected, directly or indirectly, several glycolytic enzymes [80]

Cancer	Drug development	Proteomics study showing that several metabolism-related proteins, molecular chaperons, and proteins involved in signaling are differently expressed after targeted chemotherapeutic treatment (Daunorubicin-GnRH-III Derivative Bioconjugate), leading to the conclusion that the bioconjugate exerts its cytotoxic action by interfering with multiple intracellular processes [81]

Cancer	Drug development and mechanisms of drug action	Proteomics study showing differential protein expression after treatment of Hepatocellular Carcinoma Cell Lines with Alendronate [82]
